# Postcircumcisional Ischemia of the Glans Penis Treated with Pentoxifylline

**DOI:** 10.1155/2013/278523

**Published:** 2013-02-04

**Authors:** Ersagun Karaguzel, Dogan S. Tok, Ilke O. Kazaz, Metin Gur, Fatih Colak, Omer Kutlu, Guner K. Ozgur

**Affiliations:** Department of Urology, Faculty of Medicine, Karadeniz Technical University, 61080 Trabzon, Turkey

## Abstract

Ischemia of the glans penis is a rare postcircumcision complication. We describe a four-year-old boy developing ischemia of the glans penis 48 h after circumcision. The ischemia completely resolved following treatment with iv pentoxifylline (PTX) for six days, and the patient was discharged without any problems. PTX treatment should be kept in mind as an alternative treatment modality in ischemia of the glans penis which is a serious potential post-circumcision complication.

## 1. Introduction

Circumcision is a frequent surgical procedure in modern-day urological practice. However, several mild or very severe intra- and postoperative complications can develop [1]. One such serious and rare complication is postcircumcisional ischemia of the glans penis. Possible causes are hematoma, blood vessel binding or cauterization, tight suture line, tightly wrapped bandages, use of local anesthetics containing adrenalin, and dorsal penile nerve block. We describe a very rare case of severe ischemia of the glans penis which developed after circumcision and treated with pentoxifylline (PTX).

## 2. Case Report

**Figure 1:**
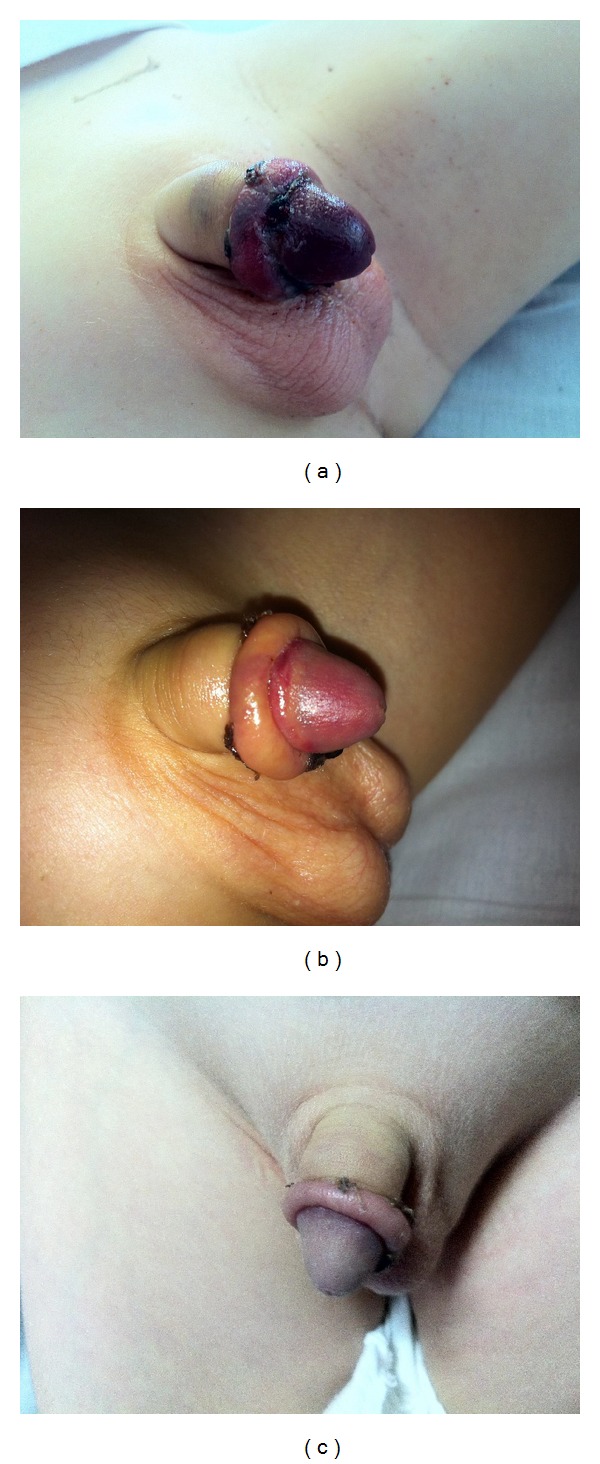
(a) Image of the glans and mucosa at initial presentation. (b) Image of the glans and mucosa after 48 hours of pentoxifylline treatment. (c) Image of the glans and mucosa entirely restored to normal at the end of day six.

Circumcision was performed on a four-year-old child under general anesthesia using bipolar cautery. No complications developed perioperatively and no local anesthetic was employed pre- or postsurgically. At 24 h routine postoperative check-up of the patient, compression bandage of the penis was removed without any other problems. At 48 h the patient represented to the emergency service with bruising and swelling in the glans and mucosa. Physical examination revealed the glanular ischemia with a black color of the mucosa and glans penis (Figure 1(a)). There were no urination problems and all laboratory findings, including whole blood count, blood chemistry, and bleeding-clotting profile were within normal limits. The patient was rehospitalized and started on iv PTX treatment at a daily dosage of 10 mg/kg divided into four equal doses. At 48 h of the treatment with PTX glanular ischemia began to decrease and the black color of the glans transformed into reddish color (Figure 1(b)). PTX treatment was maintained for six days. The color of the glans and mucosa improved slightly every day and gradually returned to normal (Figure 1(c)). Patient was discharged at the end of the sixth day. Physical examinations over the following days revealed no further problems.

## 3. Discussion

Ischemia of the glans penis is a rare condition in children. The most frequent causes are circumcision, trauma, penile strangulation, and application of vasoconstrictive agents [1–3]. Plastibell device usage, application of pre- or post-circumcision dorsal nerve block with local anesthetics containing vasoconstrictor agents like adrenaline, tight penile compression bandaging, tight suture line, and excessive use of monopolar cautery are the main reasons for the glans ischemia in circumcision [4–7]. There was no detectable reason for the ischemia of the glans in our case considering all these reasons.

The literature reports cases in which PTX was used in the treatment of post-circumcisional ischemia of the glans penis. Aslan et al. encountered a similar clinical picture in a case of ischemia of the glans on the third day after circumcision [8]. The patient was given a daily dose of 10 mg/kg iv PTX divided into four equal doses over five days. Glans resumed a normal appearance at the end of the fifth day. Tzeng et al. encountered ischemic changes in the glans penis after circumcision in a 33-year-old patient. They treated the patient with iv PTX and hyperbaric oxygen therapy and reported that the ischemia resolved completely [9].

PTX is an orally active hemorheological agent which is widely used to treat peripheral vascular and cerebrovascular diseases. It is also employed for various conditions associated with impaired regional microcirculation. It acts by raising the deformability of red blood cells and reducing blood viscosity and the potential for platelet aggregation and thrombus formation. Additionally, PTX is a powerful peripheral vasodilator. It has been shown to reduce whole blood viscosity to a significant extent, and to lower plasma viscosity by reducing plasma fibrinogen concentrations. There are no routine PTX usage indication and dosage in children. We administered a dosage that had already been employed in a previous similar case, 10 mg/kg iv PTX divided into four equal doses [8]. Forty-eight hours after the commencement of treatment the color of the glans and mucosa had decreased substantially. The color returned to normal on the sixth day.

PTX is a treatment modality with satisfying results that can be used with confidence in ischemia of the glans and mucosa which is a rare circumcision complication.
